# Asymptomatic and Pre-Symptomatic COVID-19 in China

**DOI:** 10.1186/s40249-020-00679-2

**Published:** 2020-06-22

**Authors:** Zunyou Wu, Jennifer M. McGoogan

**Affiliations:** National Center for AIDS/STD Control and Prevention, Chinese Center for Disease Control and Prevention, 155 Changbai Road, Changping District, Beijing, 102206 China

**Keywords:** Novel coronavirus disease 2019, Asymptomatic, Pre-symptomatic, Transmission

As the novel coronavirus disease 2019 (COVID-19) pandemic spreads rapidly across the globe many unanswered questions about the basic biology and epidemiology of the disease hamper our response strategies and limit our ability to achieve control and prevent a rebound or so-called “second wave”. One such crucial question is: *To what degree do asymptomatic cases contribute to transmission?* Early, small studies on this subject have found wide ranging estimates of the prevalence of asymptomatic carriers, and just a handful of studies so far have documented viral shedding by asymptomatic cases [1]. We recently re-examined China’s COVID-19 case report data to investigate this question [2]. This Editorial aims to describe how asymptomatic cases contribute to transmission and what the implications are for control strategies.

Asymptomatic COVID-19 cases are those having positive results from either viral nucleic acid or antibody testing yet not having classical symptoms (i.e., fever, dry cough, fatigue). In a report of the first 72,314 COVID-19 cases in China, the proportion of such asymptomatic cases was 1% — only 889 cases had been documented [3, 4]. However, these researchers underscored the high likelihood of this being an understatement of the true prevalence of asymptomatic infection because of the inherent difficulty of finding these cases [3, 4]. Also, it should be noted that community transmission in China was limited primarily to Wuhan City, and to a lesser extent in Hubei Province, while the 30 other provinces/municipalities/autonomous regions only had clusters of cases. The prevalence of asymptomatic cases may differ in areas with versus without community transmission. Indeed, until recently asymptomatic cases were only being found through rapid screening of close contacts of symptomatic cases, intensive investigation of case clusters, and active testing campaigns [5].

Not surprisingly, one important finding that has come to light over the past two months is that only a portion of cases in China originally categorized as asymptomatic are “true” asymptomatic cases. Even after an extended period of close medical observation, these individuals never become ill with COVID-19 symptoms, yet they eventually produce detectable levels of specific antibodies. Other individuals who have been identified as asymptomatic at their initial RT-PCR screening, were likely in the virus incubation period. Thus, they were not asymptomatic, but pre-symptomatic, and they eventually experienced the onset of symptoms, which meant that they were reclassified into one of the other case definitions (i.e., mild, moderate, severe, critical) [5].

As of April 7, 2020, a total of 81 802 COVID-19 cases had been reported in China. This total included 1190 asymptomatic cases that had been confirmed as asymptomatic after extended close follow-up. It also included a further 1095 cases that had been tentatively categorized as asymptomatic cases but were still under medical observation [6]. These findings place the prevalence of “true” asymptomatic infection in the range of 1.5 to 2.8%. Nevertheless, this is clearly still an underestimate since testing has primarily occurred among individuals who have symptoms. Interestingly, the new widespread active testing campaign currently underway in Wuhan (~11 million residents planned to be tested over 10 days) may provide important new evidence on the prevalence of carriers. Meanwhile, asymptomatic and pre-symptomatic cases are beginning to be documented in other settings as well, for example, in a long-term care facility in the United States [7].

As for the infectiousness of asymptomatic or pre-symptomatic cases, it is important to note that presence of viral RNA (i.e., positive viral nucleic acid test result) does not necessarily indicate the presence of viable, transmissible virus [8]. Yet, transmission events have been documented in various contexts in China [6], and now elsewhere as well [9], wherein asymptomatic or pre-symptomatic individuals successfully pass their infection on to close contacts. Transmission in this context is almost certainly a driver of local outbreaks and epidemics and thereby contributes to the global pandemic. The magnitude of its contribution to the epidemic was not significant in China according to the limited available data; however, it remains unknown in other countries. Large scale serological studies, which will help our understanding of transmission by carriers, are underway in China and Germany and regionally in the United States, and their results are anxiously awaited.

Despite the epidemic being under control in China, most Chinese citizens are still susceptible to COVID-19 and people are extremely concerned about a resurgence that could be sparked by undetected transmission by asymptomatic and pre-symptomatic individuals. But China is not alone. Many other countries, particularly low- and middle-income countries the world over, must also be vigilant with respect to this silent danger [10]. The stakes are high in these countries, where many live in crowded and impoverished communities and cannot easily adopt personal hygiene and social distancing measures. Moreover, the communities themselves may struggle to implement environmental disinfection procedures; testing, isolation, contact tracing, and quarantine; or engage in community containment actions. Sadly, without these nonpharmacological interventions, and without vaccines and therapeutics, and without a strong healthcare system, these communities will likely suffer the worst of what COVID-19 brings.

## Data Availability

Data sharing is not applicable to this article as no datasets were generated or analyzed during the current study.
